# Molecular Cytogenetic Characterization of the Dioecious *Cannabis sativa* with an XY Chromosome Sex Determination System

**DOI:** 10.1371/journal.pone.0085118

**Published:** 2014-01-21

**Authors:** Mikhail G. Divashuk, Oleg S. Alexandrov, Olga V. Razumova, Ilya V. Kirov, Gennady I. Karlov

**Affiliations:** Centre for Molecular Biotechnology, Russian State Agrarian University – Moscow Timiryazev Agricultural Academy, Moscow, Russia; CNRS/University Lyon 1, France

## Abstract

Hemp (*Cannabis sativa* L.) was karyotyped using by DAPI/C-banding staining to provide chromosome measurements, and by fluorescence *in situ* hybridization with probes for 45 rDNA (pTa71), 5S rDNA (pCT4.2), a subtelomeric repeat (CS-1) and the *Arabidopsis* telomere probes. The karyotype has 18 autosomes plus a sex chromosome pair (XX in female and XY in male plants). The autosomes are difficult to distinguish morphologically, but three pairs could be distinguished using the probes. The Y chromosome is larger than the autosomes, and carries a fully heterochromatic DAPI positive arm and CS-1 repeats only on the less intensely DAPI-stained, euchromatic arm. The X is the largest chromosome of all, and carries CS-1 subtelomeric repeats on both arms. The meiotic configuration of the sex bivalent locates a pseudoautosomal region of the Y chromosome at the end of the euchromatic CS-1-carrying arm. Our molecular cytogenetic study of the *C. sativa* sex chromosomes is a starting point for helping to make *C. sativa* a promising model to study sex chromosome evolution.

## Introduction

Dioecy occurs in approximately 7% of flowering plant species [1], [2], among which only a small number of species have cytogenetic and/or molecular evidence for sex chromosomes. In contrast to animals, where dioecism is often accompanied by sex chromosome dimorphism, cytogenetically distinguishable sex chromosomes have been reported only in 19 species, in 16 angiosperm families [2]. Sex chromosomes are thought to have evolved independently in plants many times, suggesting a recent origin of sex chromosome dimorphism in many plants [2], [3], [4], [5], [6], [7], [8], [9].

Few plants with sex chromosomes have yet been studied with molecular genetic tools. Cytogenetic landmarks for the sex chromosomes and fully sex-linked DNA markers have been established in species of *Rumex* [10], [11], [12], [13], [14], *Silene* [15], [16], [17], [18], [19], *Humulus* [20], [21], [22], [23], [24], [25], [26], and *Coccinia* (Cucurbitaceae) [27], but sex-linked genes have so far been identified in dioecious Silene species (mainly *S. latifolia*) [28], [29], [30], [31], [32], [33] and in papaya (*Carica papaya*) [34].

The small family Cannabaceae *sensu lato* (with 11 genera) includes the Cannabaceae *sensu stricto,* whose three species are all dioecious, and is a member of the urticalean rosids, whose other members include Urticaceae *sensu lato* (with both monoecious and dioecious species) and Moraceae (with a high proportion of dioecious species). Hemp (*Cannabis sativa*, 2n = 20) and the common hop (*Humulus lupulus*, 2n = 20) have XX/XY chromosome system, while Japanese hop (*H. japonicus,* 2n = 16 for female and 17 for male) has an XX/XY1Y2 system. *C. sativa* has a male-determining Y [2], [35], but, in *H. lupulus* and *H. japonicus*, sex is determined by the X to autosome ratio, although the Y chromosome is essential for normal pollen development in *H. lupulus* [36]. *C. sativa* has a smaller genome size (0.84–0.91 pg [37], [38]) than those of the two Humulus species (1.7 pg for *H. japonicus* [39], [40], and 2.90 pg for *H. lupulus* [39]). The karyotypes of *H. lupulus* and *H. japonicus* have been well studied. In contrast to most plants with sex chromosomes, the *H. lupulus* Y chromosome is the smallest in the karyotype [21], while in *H. japonicus* the Y1 and Y2 chromosomes are the largest in the karyotype [23].

Many male-specific DNA markers have been identified in *C. sativa,* allowing male and female plants to be identified in early developmental stages [41], [42]. However, despite recent progress in *C. sativa* genome sequencing and genomics [43], we know little about its sex chromosome structure apart from the basic karyotype information outlined above. The species’ estimated haploid genome sizes are 818 Mb for female plants and 843 Mb for males, indicating that the Y chromosome is larger than the X [37], although this difference is not usually detectable by microscopic techniques [37], Yamada, 1943, cited by [44], [45], and very precise measurements are needed to identify the Y chromosome. Karyotype analysis of DAPI stained chromosomes suggested that the X chromosome is submetacentric and the Y chromosome is subtelocentric, with a satellite at the terminus of its short arm, but Chattopadhyay (1989) [cited by 46], observed no heteromorphic bivalent during meiosis, suggesting that the chromosomes do not show strong heteropycnosis or size differences. However, it was shown that one arm of chromosome Y was heterochromatic and that its terminal region contained 100 to 200 copies of a LINE-like retrotransposon repeated sequence [47].

Modern cytogenetic methods have not previously been applied to *C. sativa* for karyotype analysis. Here, we characterize the karyotype of male and female *C. sativa* L. using DAPI banding and FISH using 5S and 45S rDNA probes, as well as probes for telomeric and subtelomeric repeats on mitotic and meiotic cell preparations. At present, no fully assembled Y chromosome from plants is available, other than the small Y region in papaya (*Carica papaya* L) [48]. The likely small size of the *C. sativa* genome (which is estimated to be about 825 Mbp) and the completion of the draft genome sequence of this species could enable this to become possible. Our results providing basic molecular cytogenetic data on the structure of the *C. sativa* genome, including the sex chromosomes, could provide a starting point for genome assembly.

## Materials and Methods

### Plant material

The following male and female *Cannabis sativa* plants were used in these experiments. For the study of mitosis, cv “Zenitsa” seedlings (P.P. Lukyanenko Krasnodar Research and the Development Institute of Agriculture, Krasnodar, Russia) were harvested. To study meiosis, young buds from the “T-80” line were provided by Dr S. Dolgov (Branch of M.M. Shemyakin and Yu. A. Ovchinnikov Institute of Bioorganic Chemistry of the RAS, Pushchino, Moscow Region, Russia).

### Mitotic chromosome preparation

Actively growing root tips approximately 1.5 – 2.0 cm long were harvested separately from young hemp seedlings and immediately pre-treated with a 2 mM aqueous solution of 8-hydroxyquinoline for 2 h at room temperature (RT) and then for 2 h at 4°C in the dark. A 3:1 ethanol/glacial acetic acid (v/v) mix was used for fixation. Meristems 2 mm long were cut from fixed root tips and digested in a 10 μl enzyme solution (0.5% cellulase Onozuka R-10 (Serva, Germany) and 0.5% pectolyase Y-23 (Seishin Corp., Japan)) in 10 mM citrate buffer (pH = 4.9) for 1.5 h at +37°C. Suspended cells were used for chromosome preparation as described by Henegariu [49] and Kato et al. [50].

### Meiotic chromosome preparation

Young buds were harvested for meiotic chromosome preparation. Screening of the meiosis stages was performed in one of five anthers by chromosome preparation with acetocarmine staining. Anthers at diakinesis were fixed in a 3∶1 ethanol and glacial acetic acid (v/v) mix. Prior to chromosome preparation, the anthers were washed in 0.5 ml of distilled water for 1 h. Washed anthers were digested in an enzyme solution (0.9% cellulase Onozuka R-10 (Serva, Germany), 0.3% pectolyase Y-23 (Seishin Corp., Japan), and 0.9% cytohelicase from *Helix pomatia* C8274 (Sigma, USA)) in 10 mM citrate buffer, pH = 4.9. Anthers were crushed in 60% acetic acid. Then, a 3∶1 mixture of ethanol and glacial acetic acid (v/v) was added to the slide around and in the center of the drop. Each slide was washed with 96% ethanol and dried.

### DNA isolation

DNA isolation was performed as described by Doyle and Doyle [51] with some modifications. The extracting buffer contained 100 mM Tris-HCl (pH = 8.0), 20 mM EDTA (pH = 8.0), 2 M NaCl, 1.5% CTAB, 1.5% PVP and 0.2% β-mercaptoethanol. A 15 mM ammonium acetate solution in 75% ethanol was used for DNA washing.

### PCR test for sex identification of plants

As chromosome preparations were performed on young seedlings, sex identification of plants was required. A PCR-test with the molecular markers MADC2 and SCAR332 was used for sex identification, as described by Mandolino et al. [41] and Torjek et al. [42]. The modified program for the MADC2 primers consisted in 94°C for 5 min, followed by 35 cycles of 94°C for 30 s, 60°C for 1 min, and 72°C for 1 min, and then 72°C for 5 min. The modified program for the SCAR332 primers consisted in 94°C for 2 min, followed by 35 cycles of 94°C for 10 s, 62.5°C for 30 s, 72°C for 1 min, and then 72°C for 2 min.

### BLAST analysis

A search for the hemp subtelomeric repeat was performed with a BLAST search (http://blast.ncbi.nlm.nih.gov) using the hop subtelomeric repeat sequence (GU831574) to probe the *Cannabis sativa* whole-genome shotgun contigs. In the search results, a contig (AGQN01004814) with one repeat of low homology to the hop subtelomeric tandem repeat was found. Nineteen tandemly repeated units about 375 bp in size from this contig were found using the GenDoc software (http://gendiapo.sourceforge.net) and were subsequently used for primer design. The primers (CS-1f 3′-GGTACCACTATGAGAAATGTGAGA-5′ and CS-1r 3′-CCTTTGTGAAATGTGGCCC-5′) used to amplify the detected repeat units were designed using PRIMER3 v.0.4.0 (http://frodo.wi.mit.edu). The PCR products from the CS-1 reaction were purified and cloned into the pGEM®-T Easy Vector A1360 (Promega, USA) in *E. coli* cells. Plasmids with inserts were isolated from cells using the GeneJET ™ Plasmid Miniprep Kit (Fermentas, Lithuania). The nucleotide sequences of the inserts were determined on an ABI3130XL instrument (Applied Biosystems, Inc., USA) after sequencing reactions were performed with a Big Dye Terminator v 1.1. Cycle Sequencing Kit (Applied Biosystems, Inc., USA).

### DNA probes and Fluorescent *in situ* hybridization (FISH)

The following probes were used: pTa71 (18S-28S rDNA) [52], pCT4.2 (5S rDNA) [53], the *Arabidopsis*-type telomere probe (5'-CCCTAAA-3')_3_ synthesized with a TAMRA label (ZAO “Syntol”, Moscow, Russia) and the CS-1 probe (*C.sativa* subtelomeric repeat (JX402748)). All DNA probes were labeled by nick translation and PCR according to the manufacturer’s instructions (Boehringer, Germany) with biotin-16-dUTP (CS-1, pCT4.2) or digoxigenin-11-dUTP (pTa71). FISH experiments were performed as described by Karlov et al. [21]. The chromosomes were counterstained with 1 mg/ml DAPI and mounted in Vectashild (Vector laboratories, UK). An AxioImager M1 fluorescent microscope (Zeiss) was used to observe chromosome preparations. The metaphase plates with fluorescent signals were photographed with a monochrome AxioCam MRm CCD camera and visualized using Axiovision software (Zeiss). Metaphase chromosomes were classified according to Levan et al. [54] based on their arm ratio and FISH hybridization pattern. In each experiment, at least 20 mitotic metaphase plates from each of the 11 male and 8 female plants were analyzed.

## Results

### Karyotype

**Figure 1:**
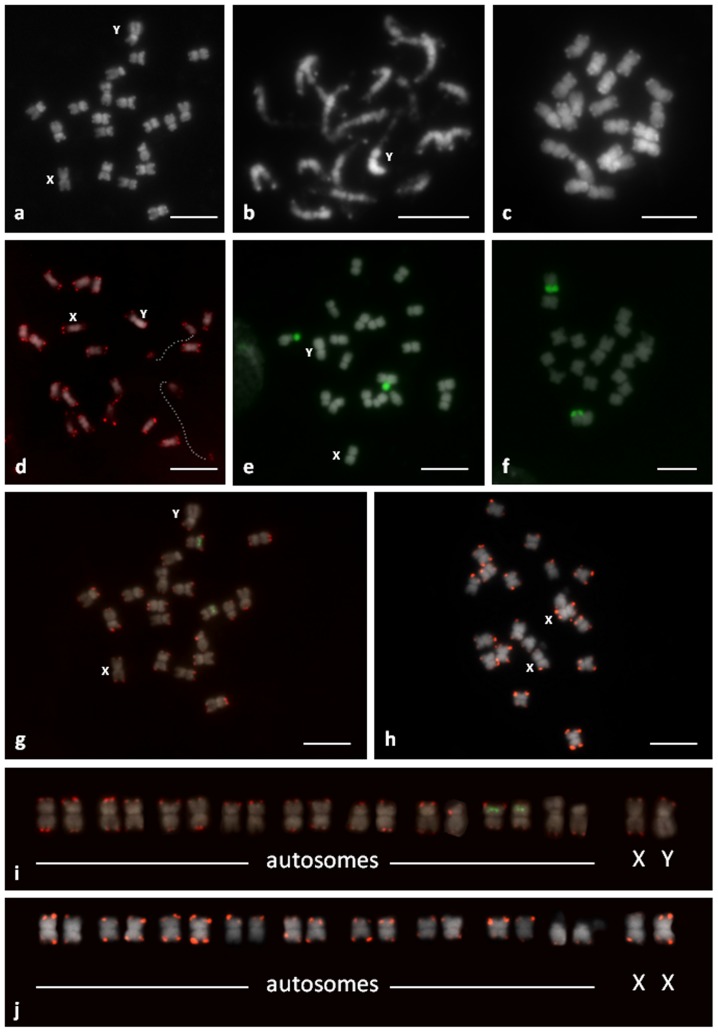
Chromosomes of *C. sativa*: C-banding/DAPI, male metaphase (a), male prometaphase (b) and female metaphase (c). Distribution of *Arabidopsis-*type (5′-CCCTAAA-3′) telomeric sequences (small red dots located at the end of the chromosomes) (d). FISH with 45S rDNA (green signals) on male (e) and female (f) metaphase chromosomes. Bicolor FISH to male metaphase (g) with CS-1 subtelomeric repeat (red) and 5S rDNA probe (green). FISH to female metaphase (h) with CS-1 subtelomeric repeat (red). The karyotypes of male (i) and female (j) plants. Bar  =  5 µm.

The metaphase cells in both female and male root tips showed many polyploid cells. The chromosomes are small, and varied from 2.6 to 3.8 µm, and could not all be distinguished by their length and centromere position. The autosome complement consisted of 8 pairs of metacentric (m) chromosomes and one pair of satellite (SAT) NOR (nucleolus organizer region) - bearing submetacentric chromosomes (sm), ordered in pairs 1–9 according to their length and arm ratio (Fig. 1). The haploid karyotype formula of *C. sativa,* describing number of each chromosome types, was 8m + 1sm (SAT) + Xm/Ym for male and 8 m + 1sm (SAT) + Xm for female plants.

### DAPI

Due to the small size of these chromosomes, it was difficult to obtain high-quality DAPI banding. DAPI-positive bands were seen at the ends of all chromosomes, but not on all arms (Fig. 1a-c). Weak DAPI-positive bands were also detected near the centromere of one pair of NOR-bearing chromosomes and one pair of metacentric chromosomes. In the prometaphase chromosomes, pericentromeric heterochromatin and proximal euchromatin was also visible (Fig. 1b). In male plants, one of the largest chromosomes showed brighter DAPI staining on one heterochromatic arm relative to the other euchromatic arm of the chromosome (Fig. 1a, b). This chromosome was likely the Y chromosome. The second largest chromosome without homologous DAPI positive bands on both subtelomeres was probably the X chromosome. A pair of these chromosomes was detected in female plants.

### FISH

*C. sativa* chromosomes were further analyzed by FISH using repetitive DNA probes. Arabidopsis-type telomeric repeats (5′-CCCTAAA-3′) were observed on the termini of all *C. sativa* chromosomes (Fig. 1d), and no interstitially located signals from telomeric sequences were observed in the material analyzed. The subtelomeric repeat probe (CS-1) showed clear signals in the subtelomeric regions of both arms of all chromosomes in male and female plants, except for the heterochromatic arm of chromosome Y, the NOR-bearing arm of autosomes (see below) and the long arm of some other autosome pairs (Fig. 1g, h). The DAPI positive subtelomeric bands colocalized with CS-1.

FISH with the pTa71 (18S-5,8S-25S rDNA) probe revealed a signal on the NOR-bearing arm of the single chromosome pair of male and female plants (Fig. 1e, f). The size of the signal differed between homologous chromosomes. The pCT4.2 (5S rDNA) probe detected the pericentromeric position of another autosome pair, which carried the CS-1 subtelomeric repeat on both chromosome arms (Fig.1g). Based on these data, the karyotype of male and female plants was constructed (Fig.1i. j).

**Figure 2:**
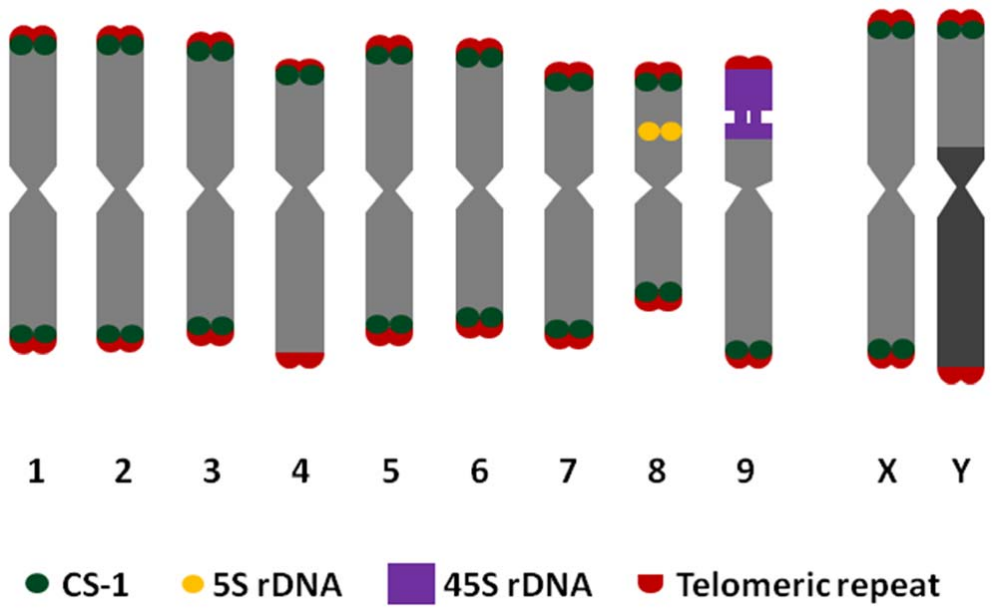
Idiogram of haploid chromosome complement of *C. sativa,* including *Arabidopsis-*type telomeric repeat, CS-1 subtelomeric repeat, 45S rDNA and 5S rDNA sites.

The Y chromosome can be distinguished from all the autosomes by its larger size, the presence of a fully heterochromatic arm and the presence of CS-1 only on the euchromatic chromosome arm (stained less intensely with DAPI, Fig. 2). The largest chromosome was assumed to be the X chromosome, and carries CS-1 subtelomeric repeats on both arms. No morphological or significant size differences were detected between the X chromosome and the autosomes (Fig. 2), and identification of the X on metaphase plates was problematic. The X chromosome was not clearly differentiated from the autosomes because they have a similar size (table 1) and the presence of CS-1 at terminal positions on both arms (Fig. 2). Very precise measurements are needed to identify the X chromosome. Three of the nine pairs of autosomes could be distinguished individually using pTa71 (18S-5, 8S-25S rDNA), pCT4.2 (5S rDNA) and CS-1 probes.

**Table 1:** Size of sex chromosomes compared to the largest autosome.

Chromosome	Relative chromosome length, %
Y	6.5±0.18
X	6.1±0.19
The largest autosome	5.6±0.12

### Meiotic observation

**Figure 3:**
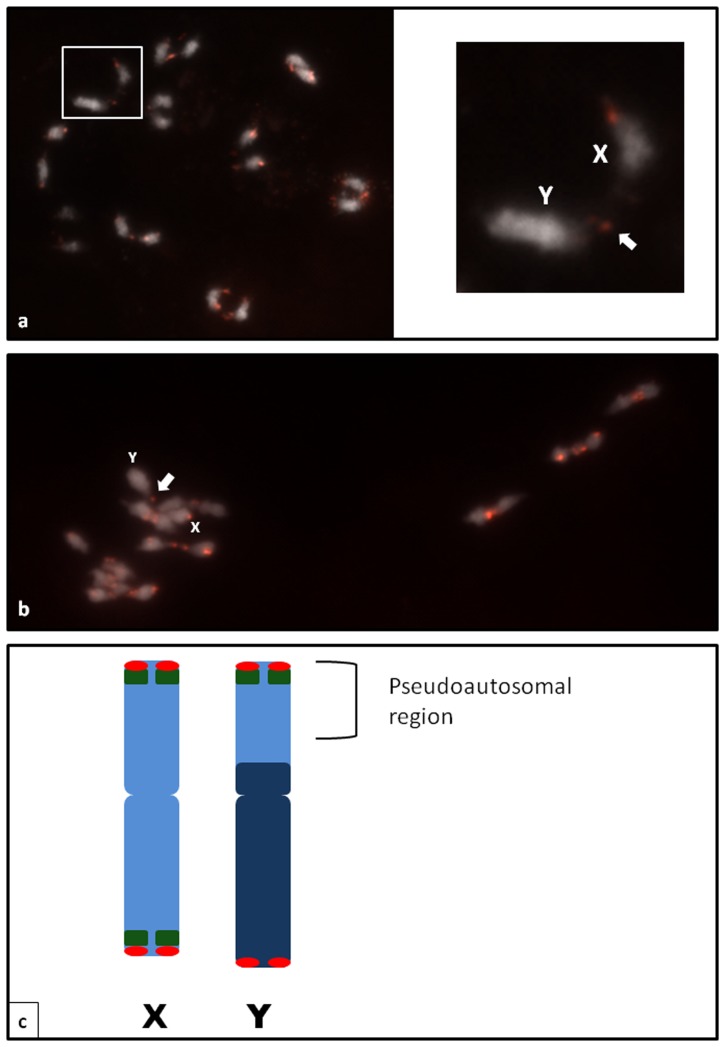
The meiotic chromosomes of *C. sativa* at diakinesis (a) and metaphase I (b). The chiasma between the sex chromosomes can clearly be seen and indicated by arrows. **c** Idiogram of the *C. sativa* XY chromosomes with the hybridization sites of CS-1 (green) and the *Arabidopsis-*type telomeric repeat (red). The pseudoautosomal region is indicated by brackets.

The meiotic chromosome complement of *C. sativa* consisted of ten similarly sized bivalents, and FISH analysis with the CS-1 probe in the diakinesis stage confirmed the mitotic chromosomal observations. The XY open bivalent of male plants had signals in the same positions as in mitotic chromosomes (Fig.3a, b). The telomeric chiasma of the open sex bivalent was found to occur between the Y chromosome euchromatic arm carrying the CS-1 subtelomeric repeat and one arm of the X chromosome, which possessed CS-1 on both arms. In all meiotic chromosome complements studied, similar chiasmata were observed on the sex chromosomes pair. We therefore conclude that the pseudoautosomal region (PAR) of the Y chromosome consists of the distal parts of the euchromatic arm (Fig. 3c).

### Chromosome distribution of rDNA, subtelometic repeat and PAR in Cannabaceae

**Figure 4:**
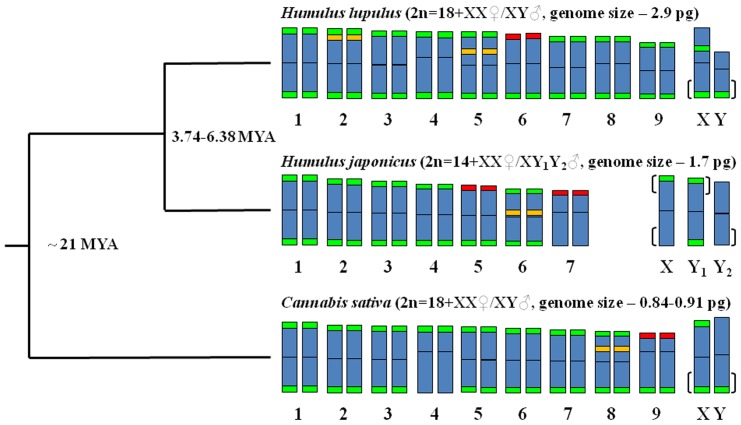
Simplified phylogeny of Cannabaceae genus included in idiograms. The phylogeny is according to the reference of [59]. Idiograms created based on data obtained in [21], [23], [24], [26], [55] and in this study. 5S rDNA: green signals; 45S rDNA: red signals; species-specific subtelomeric repeats (HSR-1for *H. lupulus*, HJSR for *H. japonicus* and CS-1 for *C. sativa*): green signal. The position of pseudoautosomal region on sex chromosomes is indicated by brackets. Time of divergence estimated in [60], [61], [62].

In order to compare and analyze the evolutionary relationships among *Humulus lupulus, H. japonicus* and *Cannabis sativa* the PAR localization on sex chromosomes, chromosomal distribution of 5S and 45S rDNA, as well as subtelomeric species specific DNA repeats based on data obtained in this study and presented in previous papers [21], [23], [24], [26], [55] are summarized and displayed schematically in Figure 4. The number of 5S and 45 rDNA loci differs among these species. The main difference in subtelomeric repeat location was detected on sex chromosomes, especially in *H.lupulus* where this repeat was hybridized near centromere position on X chromosome. The location of PAR was in agreement with subtelomeric repeat location on X and Y chromosomes.

## Discussion

FISH performed with 5S and 45S rDNAs probes differentiated two pairs of *C. sativa* autosomes from the other chromosomes and from each other. In addition to the considerably smaller nuclear DNA amount than in the closest relatives of this species (suggesting extensive DNA/chromatin elimination or accumulation of repetitive sequence), and in chromosomes counts (see above), the karyotypes of the perennial *H. lupulus* and annual *H. japonicus* also differ (Fig.4). A first difference is the location of rDNA. In *H. lupulus*, two chromosomes carry 5S rDNA loci and one carries the 45S rDNA locus, whereas in *H. japonicus* one chromosome carries the 5S rDNA and two carry the 45S rDNA loci [21], [23], [55]. However, the structural organization of the nuclear ribosomal RNA genes of all three species is similar [56]. The differences in the number of rDNA sites and chromosome counts suggest that chromosome translocations must have occurred.

Under the most widely accepted theory of sex chromosome evolution, the X and Y were derived from a pair of autosomes that became differentiated after suppressed recombination evolved around the locus controlling sex determination [2], [4], [7], [58], [59]. Among the events and processes that may have been involved in suppressing recombination between the Y and X chromosomes are chromosome rearrangements [49], [59]. The distribution of signals using probes for species-specific subtelomeric repeats in *H. lupulus* (HSR-1) [24], *H. japonicus* (HJSR) [26] and *C. sativa* (CS-1, this study) showed the following evidence suggesting chromosome rearrangements, particularly in the sex chromosomes. The *C. sativa* Y chromosome has CS-1 repeats at the end of only one chromosome arm, and this is similar to HSR-1 in *H. lupulus*. The X chromosomes of both *C. sativa* and *H. lupulus* have two sites with subtelomeric repeats, but in *H. lupulus* the second site is placed interstitially near the centromere of the chromosome. In *H. japonicus*, the HJSR located only on one X chromosome arm, but the Y1 chromosome has repeats on both arms, while Y2 does not have subtelomeric repeats. The orientation of the pseudoautosomal regions on the X and Y (or Y1 and Y2) chromosomes indicates an important role of subtelomeric repeats in sex chromosome genesis. We found Arabidopsis-type telomeric repeats at the termini of all hemp chromosomes, as in *H. japonicus* and *H. lupulus* [24], [26]. Because we found no interstitial telomeric FISH signals in *C.sativa*, no long telomeric sequence can have been involved in translocations or inversions in hemp.

The *C. sativa* Y chromosome arm that does not pair with the X chromosome is heterochromatic and is visibly more condensed compared with pairing region. The Y1 and Y2 chromosomes of *H.japonicus* are also heterochromatic and showed distinctly stronger DAPI fluorescence [23]. In *S. latifolia*, the non-pairing region of the Y chromosome appears relatively condensed [8], possibly due to high level of DNA methylation or/and the accumulation of repetitive sequences such as retrotransposons. Accumulation of repetitive sequences on Y chromosomes has been detected in studies of species of *Rumex acetosa* [11], *Silene latifolia* [18] and *C. sativa* [47]. The DAPI positive staining of the Y chromosome arm in this study suggests that it may be enriched in repeated sequences.

In *H. lupulus* the Y is the smallest chromosome, while in *H. japonicus* the Y1 and Y2 are the largest chromosomes [21], [23], [24], [26]. Our results show that the Y is larger than the autosomes and the X chromosome in *C. sativa*. The small size of *H. lupulus* Y probably due to that it has undergone deletion of parts that are present in the Ys of *H. japonicus* and *C.sativa*. The polymorphic sex chromosome system in *H. japonicus* could have arisen by the translocation of an autosome to the X chromosome and the homologous (nontranslocated) autosome becomes a second Y chromosome [23]. The limited data available so far reveal no relationship between the ages of sex chromosomes and the extent of Y/autosome or X/Y divergence and also suggest that transposon accumulation and chromosome rearrangements occur idiosyncratically [27]. All these observations suggest that the paths of plant sex chromosomes evolution are not uniform. It is therefore of interest to test whether the *C. sativa* X and Y are homologous with the X and/or Y of the related species. However, this is not yet possible, as none of our probes are specific for a single-copy sequence and it is a task for the future research.
